# Effects of External Radiation Exposure on Perinatal Outcomes in Pregnant Women After the Fukushima Daiichi Nuclear Power Plant Accident: the Fukushima Health Management Survey

**DOI:** 10.2188/jea.JE20210252

**Published:** 2022-12-05

**Authors:** Shun Yasuda, Kanako Okazaki, Hironori Nakano, Kayoko Ishii, Hyo Kyozuka, Tsuyoshi Murata, Keiya Fujimori, Aya Goto, Seiji Yasumura, Misao Ota, Kenichi Hata, Kohta Suzuki, Akihito Nakai, Tetsuya Ohira, Hitoshi Ohto, Kenji Kamiya

**Affiliations:** 1 Department of Obstetrics and Gynecology, Fukushima Medical University School of Medicine, Fukushima, Japan; 2 Radiation Medical Science Center for the Fukushima Health Management Survey, Fukushima Medical University, Fukushima, Japan; 3 Department of Physical Therapy, Fukushima Medical University School of Health Sciences, Fukushima, Japan; 4 Department of Epidemiology, Fukushima Medical University School of Medicine, Fukushima, Japan; 5 Center for Integrated Science and Humanities, Fukushima Medical University, Fukushima, Japan; 6 Department of Public Health, Fukushima Medical University School of Medicine, Fukushima, Japan; 7 Department of Midwifery and Maternal Nursing, Fukushima Medical University School of Nursing, Fukushima, Japan; 8 Fukushima Society of Obstetrics and Gynecology, Fukushima, Japan; 9 Department of Health and Psychosocial Medicine, Aichi Medical University School of Medicine, Aichi, Japan; 10 Nippon Medical School Tamanagayama Hospital, Tokyo, Japan; 11 Research Institute for Radiation Biology and Medicine, Hiroshima University, Hiroshima, Japan

**Keywords:** nuclear accident, radiation dose, maternal exposure, pregnancy, perinatal outcomes

## Abstract

**Background:**

This study aimed to investigate the effects of maternal exposure to external radiation on perinatal outcomes among women who experienced the Fukushima Daiichi Nuclear Disaster (FDND) using the Fukushima Health Management Survey (FHMS).

**Methods:**

Data from the Pregnancy and Birth Survey and Basic Survey in the FHMS were combined to analyze external maternal radiation exposure following the FDND, and the relationship between radiation dose and perinatal outcomes was analyzed using binomial logistic regression analysis. Missing dose data were supplemented using multiple imputation.

**Results:**

A total of 6,875 individuals responded to the survey. Congenital anomalies occurred in 2.9% of patients, low birth weight (LBW) in 7.6%, small for gestation age (SGA; <10th percentile) in 8.9%, and preterm birth in 4.1%. The median maternal external radiation dose was 0.5 mSv (maximum, 5.2 mSv). Doses were classified as follows: <1 mSv (reference), 1 to <2 mSv, and ≥2 mSv. For congenital anomalies, the crude odds ratio for 1 to <2 mSv was 0.81 (95% confidence interval [CI], 0.56–1.17) (no participants with congenital anomaly were exposed to ≥2 mSv). At 1 to <2 mSv and ≥2 mSv, the respective adjusted odds ratios were 0.91 (95% CI, 0.71–1.18) and 1.21 (95% CI, 0.53–2.79) for LBW, 1.14 (95% CI, 0.92–1.42) and 0.84 (95% CI, 0.30–2.37) for SGA, and 0.91 (95% CI, 0.65–1.29) and 1.05 (95% CI, 0.22–4.87) for preterm birth.

**Conclusion:**

External radiation dose due to the FDND was not associated with congenital anomalies, LBW, SGA, or preterm birth.

## INTRODUCTION

The tsunami caused by the Great East Japan Earthquake of March 11, 2011 directly hit the Fukushima Daiichi Nuclear Power Plant, causing the Fukushima Daiichi Nuclear Disaster (FDND), which resulted in fear of fetal radiation exposure and subsequent evacuation, forcing pregnant women to change medical institutions for prenatal check-ups and resulting in considerable physical and mental stress.^1^

Fukushima Prefecture launched the Fukushima Health Management Survey (FHMS) in July 2011 to monitor the future health of prefectural residents by assessing the direct damage caused by the disaster and the FDND radiation effects.^2^^,^^3^

FHMS comprises a detailed and Basic Survey (BS). The BS covered all residents of Fukushima Prefecture between March 11 and July 1, 2011 and estimated the external radiation doses in the 4 months following FDND, obtaining baseline data for health monitoring and protection. The Pregnancy and Birth Survey (PBS), a questionnaire-based survey, was commissioned by Fukushima Prefecture and conducted by Fukushima Medical University to assess the health status of pregnant and nursing mothers in Fukushima Prefecture who may have been forced to evacuate or change medical facilities.^4^

The PBS provides data on perinatal outcomes, including preterm birth, low birth weight (LBW), small for gestational age (SGA), congenital anomalies,^5^^–^^9^ postpartum depression,^10^^,^^11^ hypertensive disorders of pregnancy (HDP),^12^ and the impact of the earthquake on newborns.^13^ Rates of stillbirth, preterm birth, LBW, and congenital anomalies did not deviate from the Japanese standard frequency,^1^^,^^5^ and SGA incidence was not elevated by the earthquake or its aftermath.^8^ Depressive symptoms were observed in some new mothers in Fukushima Prefecture,^10^ particularly those living near the power plant or who experienced miscarriage or stillbirth.^11^ Pregnant women living near the earthquake epicenter during their third trimester were at a higher risk for HDP, suggesting an association with psychological stress.^12^ However, no regional differences in the mothers’ and infants’ conditions were observed at 1-month postnatal check-ups.^13^

The negative effects of fetal radiation exposure, transgenerational effects of radiation exposure, and effects on germline de novo mutation have been elucidated.^14^^–^^18^ Radiation exposure following the Chernobyl accident did not change the frequency of fetal malformations,^14^ and no fetal effects or association with maternal exposure were observed among those who survived the Hiroshima and Nagasaki bombings.^16^ Similarly, low-dose exposure following FDND is considered free from effects, but no studies have examined associations with actual exposure dose.

Here, we investigated the relationship between maternal external radiation dose and major PBS outcomes—congenital anomalies, LBW, SGA, and preterm birth—based on individuals’ external radiation doses. Few reports have examined these relationships, and our results will impact disaster medicine greatly.

## METHODS

### Study population and design

In Japan, pregnant women receive a Maternal and Child Health Handbook from their municipality when they register their pregnancy and receive free antenatal and infant health check-ups. The PBS, a cohort study and a division of FHMS, was conducted as follows: fiscal year (FY) 2011 PBS questionnaires were sent to women who received a handbook from municipalities in Fukushima Prefecture between August 1, 2010, and July 31, 2011, and were completed voluntarily and returned by mail. This practice continued subsequently, with online responses introduced in 2016. Subjects in the FY2011 survey were defined as Group A if March 11, 2011, fell between 2w0d of pregnancy, which coincides with the time of fertilization, and the delivery date. Respondents from FY2011 whose 2w0d of pregnancy fell after March 11, 2011, and respondents between FY2012 and FY2018 were designated as Group B. Participants living outside the prefecture when the disaster occurred were excluded, as were those with multiple pregnancies and pregnancy termination at <22 weeks’ gestation. Infants with congenital anomalies, stillbirth, or missing birth weight and maternal parity data were excluded.

### Primary outcomes

The primary outcomes of this study were perinatal outcomes, namely congenital anomalies, LBW, SGA, and preterm birth. A congenital anomaly was defined as any indication of congenital anomaly in the questionnaire. We excluded cases with missing data on the following congenital anomalies: cataract, heart, kidney, or urinary tract anomaly, neural tube defects, microcephaly, hydrocephaly, cleft lip or palate, digestive tract atresia, imperforate anus, polydactyly, syndactyly, or other (with free response). Stillbirths and cases with missing data regarding gestational age at delivery were also excluded. LBW was defined as birth weight <2,500 g, excluding infants with congenital anomalies, stillbirth, or missing birth weight data. SGA was defined as birth weight <10th percentile for the gestational age based on the child’ sex and mother’s parity.^19^ Preterm birth was defined as delivery between 22 and 37 weeks’ gestation.

### Factors associated with the primary outcomes

The primary variable was maternal external radiation dose. In the BS, we implemented a precise system to estimate external radiation dose using technical support from the National Institute of Radiological Sciences. Self-administered questionnaires were mailed to 2,055,257 residents living in Fukushima Prefecture at the time of FDND to obtain information regarding their residence, places visited, time spent indoors and outdoors, and travel time in the 4-month period between March 11 and July 11, 2011, when atmospheric radiation levels peaked.^20^^,^^21^ Returning the survey was optional. Respondents’ behavior and location (“trail”) were used to estimate their external radiation dose in a manner similar to studies on the Chernobyl accident.^22^ The BS response rate was 27.7%, and the maternal external radiation dose distribution was confirmed using BS results; these values were then categorized. External radiation dose data were supplemented for PBS respondents who did not respond to the BS. We used multiple imputation by chained equation with predictive mean-matching methods under fully conditional specification to generate 10 datasets with imputation for the missing estimated external radiation dose.^23^^–^^27^

Other explanatory variables included maternal parity and child’s sex, height, and weight. Maternal age was defined as that on April 1 of the year following the survey. Stillbirth was defined as delivery of a dead fetus after 22 weeks’ gestation. Options for mode of pregnancy were “natural pregnancy,” “induced ovulation,” “artificial insemination with husband’s semen,” and “assisted reproductive technology.” All options, except “natural pregnancy,” were considered infertility treatments. Trimester of pregnancy at the time of the earthquake (first, second, or third) was regarded as a variable. As pregnancy complications, HDP and placenta previa were entered as outcome-related variables. HDP was defined as indicating pre-pregnancy hypertension and the development of hypertensive disease after pregnancy in the questionnaire. Placenta previa was considered present in participants who stated that they had the disease. Mental disorders were considered pregnancy complications and defined as a pre-pregnancy history of mental illness or insomnia, anxiety, and other mental disorders during pregnancy.

After FDND, the government ordered residents of 12 municipalities to evacuate.^2^ “Forced to change health check-up facility or intervals between visits due to disaster” was defined as responding “No” to the questions “Did you continue to have your antenatal check-ups and delivery at the facility where you had originally planned?” and “Were you able to receive a prenatal check-up as scheduled?” or the answer “I had to change to a facility outside of the prefecture by myself (not on the doctor’s instruction)”.

### Statistical analysis

For each major outcome, two-group comparisons were performed using the *t*-test for continuous variables and the chi-square or Fisher’s exact probability test for categorical variables (proportion and frequency). Significance was set at 5% was set using two-sided probability. Statistically significant variables were used in univariate logistic regression analysis. Fetal height was excluded as it associated with fetal weight and duration of pregnancy. To examine the association between primary outcomes and external radiation, variables found to differ significantly in the univariate analysis were employed in the multivariate binomial logistic regression analysis except as follows: Only univariate analysis was performed during congenital anomaly analysis as no covariates were suitable for multivariate analysis. Congenital anomalies include various diseases often accompanied by smaller body size, shorter gestation period, and stillbirth; therefore, gestational days at delivery, LBW, SGA, and preterm birth were not included in the multivariable analysis. Items not biologically associated with the occurrence of congenital anomalies, such as changes in health check-up facility and mental disorders during pregnancy, were also excluded. Regarding other multivariate analysis models, birth weight was strongly associated with pregnancy duration, and these items define the outcomes; therefore, we did not employ them as independent variables. The crude odds ratio and 95% confidence interval (CI) are shown for univariate analysis, and the adjusted odds ratio (aOR) and 95% CI are shown for the multivariate analysis.

Statistical analyses were performed using SAS (ver. 9.4; SAS Institute, Cary, NC, USA).

### Ethical considerations

The ethics committee of Fukushima Medical University approved this study (No. 1317, 2020-203), which was conducted in accordance with The Code of Ethics of the World Medical Association (Declaration of Helsinki) for experiments involving humans. The survey aims were detailed in a cover letter attached to the questionnaires sent to the participants. Participants were considered to provide informed consent by responding to the survey, as it was optional.

## RESULTS

**Figure 1.**
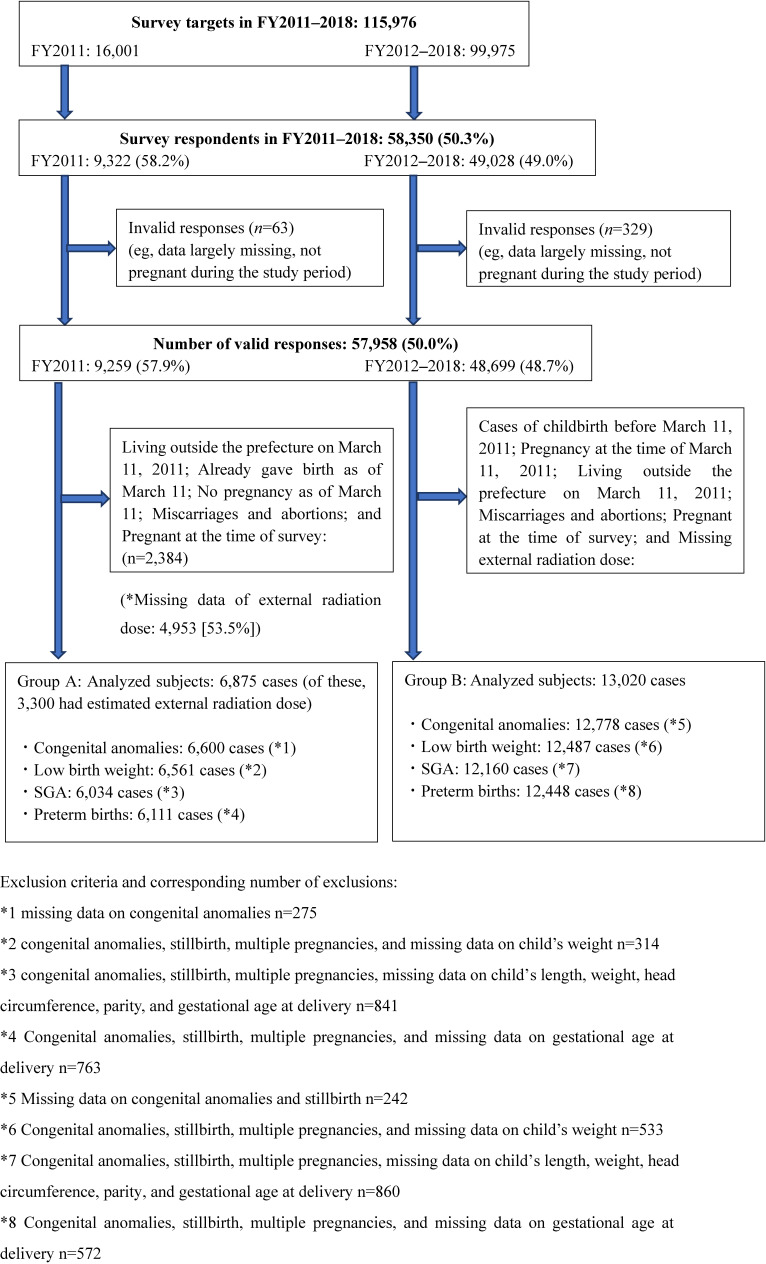
Participants of the pregnancy and birth and basic surveys

Figure 1 shows the flowchart of the process by which the survey targets of FY2011 and FY2012–2018 were selected for Group A and Group B analysis. A total of 16,001 questionnaires were mailed during FY2011. Of the 9,322 responses, 9,259 were valid (57.9%). External radiation dose data were missing in 4,953 (53.5%). After exclusion, 6,875 participants were eligible for imputation and analysis. Of these, 6,600, 6,561, 6,034, and 6,111 participants were eligible for analysis due to congenital anomalies, LBW, SGA, and preterm birth, respectively.

**Figure 2.**
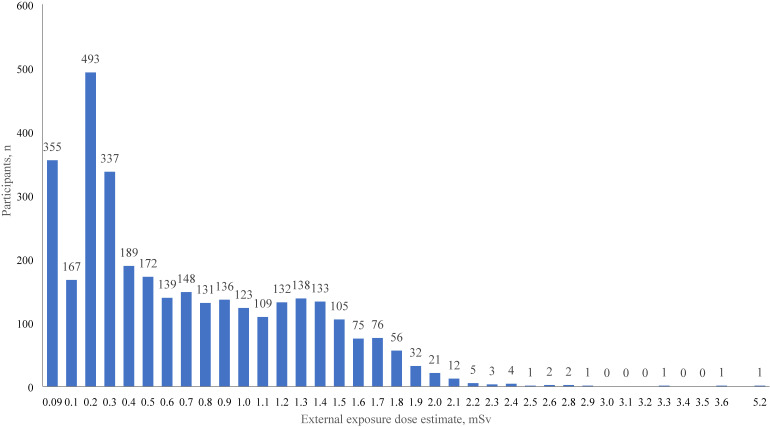
Distribution of external exposure dose estimates (*n* = 3,300)

The distribution of the external exposure dose estimates for 3,300 participants before imputation (∼48%) is shown in Figure 2. The median and mean doses were 0.5 mSv and 0.7 mSv. The highest recorded dose was 5.2 mSv. Only 1.6% of participants were exposed to ≥2 mSv radiation. The participants were therefore grouped as follows: <1 mSv (0 to <1 mSv), 1 to <2 mSv, and ≥2 mSv.

Table 1 shows the participants’ characteristics and their external radiation dose. The external exposure doses were as follows: <1 mSv, 2,267 participants (33.0%); 1 to <2 mSv, 979 (14.2%); ≥2 mSv, 54 (0.8%). Participants with missing dose data (3,575 participants, 52.0%) were classified into a single category. Rates of congenital anomaly, LBW, SGA, and preterm birth following maternal external radiation dose ≥2 mSv were 0%, 9.3%, 4.4%, and 4.3%, respectively. The relationship between maternal external radiation dose and the presence or absence of congenital anomalies, LBW, SGA, or preterm birth remains unclear (Table 2).

**Table 1.** Characteristics of 6,875 cases according to external radiation dose

	External radiation dose, mSv					
	Total		(missing)	<1 mSv	1 to <2 mSv	≥2 mSv
	Ñ	6,875 (100.0)	3,575 (52.0)	2,267 (33.0)	979 (14.2)	54 (0.8)
Maternal age, years	6,875	30.9 (5.0)	30.3 (5.2)	31.5 (4.6)	31.6 (4.7)	30.5 (5.4)
Child’s sex (male), %	6,814	51.3	51.8	50.6	51.8	46.3
Child’s length, cm	6,783	49.1 (2.2)	49.1 (2.3)	49.1 (2.2)	49.2 (2.2)	49.1 (2.1)
Child’s weight, g	6,815	3,029.3 (403.1)	3,025.6 (411.8)	3,036.1 (394.9)	3,028.0 (391.1)	3,006.2 (382.4)
Gestational days at delivery, days	6,348	276 (11.0)	275.2 (11.4)	275.7 (10.6)	276.1 (10.0)	275.3 (10.7)
Low birth weight (<2,500 g), %	6,815	7.6	7.8	7.3	7.3	9.3
SGA (<−10%), %	6,270	8.9	8.5	8.8	10.7	4.4
Congenital anomalies, %	6,600	2.9	3.1	2.9	2.0	0.0
Stillbirth, %	6,875	0.2	0.3	0.2	0.1	0.0
Preterm birth (<37 weeks), %	6,348	4.1	4.5	3.6	3.5	4.3
Multiple pregnancies, %	6,872	0.9	1.1	0.6	0.8	0.0
Primiparous, %	6,840	25.4	24.8	25.3	27.4	37.0
Infertility treatment, %	6,875	4.9	4.2	5.6	6.0	3.7
Placenta previa, %	6,875	1.4	1.3	1.3	2.0	1.9
Forced to change health check-up facility, %	6,809	35.4	32.4	45.6	23.7	20.4
Trimester of pregnancy at earthquake	6,259					
First (2–14 weeks), %		32.7	35.0	30.2	30.5	23.4
Second (14–28 weeks), %		40.0	40.3	40.5	37.1	53.2
Third (≥28 weeks), %		27.3	24.7	29.3	32.4	23.4
Hypertensive disorders of pregnancy, %	6,875	3.3	3.3	3.4	2.8	5.6
Mental disorders before birth, %	6,875	6.0	5.8	6.1	6.7	3.7
Evacuation area, %	6,875	10.8	9.0	15.5	6.5	5.6

SGA, small for gestational age.

**Table 2.** Factors associated with preterm birth, low birth weight, SGA, and congenital anomalies

	Congenital anomaly (*n* = 6,600)			Low birth weight (*n* = 6,561)		
	No	Yes	*P* ^*^	≥2,500 g	<2,500 g	*P* ^*^
	*N* (%) or mean (SD)			*N* (%) or mean (SD)		
	6,411 (97.1)	189 (2.9)		6,116 (93.2)	445 (6.8)	

External radiation dose			0.188			0.811

Missing	3,308 (51.6)	106 (56.1)		3,150 (51.5)	235 (52.8)	
<1 mSv, %	2,124 (33.1)	64 (33.9)		2,032 (33.2)	141 (31.7)	
1 to <2 mSv, %	925 (14.4)	19 (10.1)		885 (14.5)	64 (14.4)	
≥2 mSv, %	54 (0.8)	0 (0.0)		49 (0.8)	5 (1.1)	
Maternal age, years	30.9 (5.0)	30.6 (5.2)	0.456	30.9 (5.0)	31.0 (5.2)	0.458
Child’s sex (male), %	3,284 (51.3)	105 (55.9)	0.221	3,168 (51.9)	183 (41.1)	<0.001
Child’s length, cm	49.2 (2.1)	48.5 (3.4)	0.007	49.5 (1.8)	45.5 (2.8)	<0.001
Child’s weight, g	3,036 (392.5)	2,904 (556.8)	0.002	3,097 (327.5)	2,251 (318.2)	<0.001
Gestational days at delivery, days	275.7 (10.3)	272.2 (18.4)	0.013	277.0 (8.3)	260.6 (18.9)	<0.001
Primiparous, %	1,628 (25.5)	44 (23.4)	0.511	1,537 (25.3)	129 (29.1)	0.077
Low birth weight (<2,500 g), %	459 (7.2)	33 (17.7)	<0.001	—	—	
SGA, %	520 (8.8)	24 (13.9)	0.022	290 (5.2)	228 (54.0)	<0.001
Stillbirth, %	5 (0.1)	3 (1.6)	0.001^**^	—	—	
Preterm birth (<37 weeks), %	217 (3.6)	22 (12.6)	<0.001	81 (1.4)	130 (30.7)	<0.001
Forced to change health check-up facility, %	2,234 (35.2)	80 (42.6)	0.038	2,117 (35.0)	156 (35.3)	0.831
Trimester of pregnancy at earthquake			0.550			0.656
First (2–14 weeks), %	1,900 (32.3)	61 (36.3)		1,830 (32.7)	128 (30.8)	
Second (14–28 weeks), %	2,355 (40.1)	63 (37.5)		2,229 (39.8)	174 (41.9)	
Third (≥28 weeks), %	1,625 (27.6)	44 (26.2)		1,537 (27.5)	113 (27.2)	
Infertility treatment, %	316 (4.9)	12 (6.4)	0.376	275 (4.5)	30 (6.7)	0.030
Placenta previa, %	94 (1.5)	1 (0.5)	0.286	85 (1.4)	11 (2.5)	0.066
Hypertensive disorders of pregnancy, %	202 (3.2)	5 (2.7)	0.694	164 (2.7)	46 (10.3)	<0.001
Mental disorders before birth, %	382 (6.0)	18 (9.5)	0.043	368 (6.0)	24 (5.4)	0.592
Evacuation area, %	686 (10.7)	20 (10.6)	0.959	657 (10.7)	44 (9.8)	0.573

	SGA (*n* = 6,034)			Preterm birth (*n* = 6,111)		
	≥−10%	<−10%	*P* ^*^	≥37 weeks	<37 weeks	*P* ^*^
	5,516 (91.4)	518 (8.6)		5,899 (96.5)	212 (3.5)	

External radiation dose			0.103			0.335

Missing	2,854 (51.7)	252 (48.7)		3,029 (51.4)	122 (57.6)	
<1 mSv, %	1,818 (33.0)	170 (32.8)		1,954 (33.1)	60 (28.3)	
1 to <2 mSv, %	800 (14.5)	94 (18.2)		871 (14.8)	28 (13.2)	
≥2 mSv, %	44 (0.8)	2 (0.4)		45 (0.8)	2 (0.9)	
Maternal age, years	30.9 (5.0)	30.5 (5.2)	0.105	30.8 (5.0)	31.6 (5.4)	0.040
Child’s sex (male), %	2,831 (51.3)	248 (47.9)	0.134	2,983 (50.7)	120 (56.9)	0.080
Child’s length, cm	49.4 (2.0)	47.1 (2.3)	<0.001	49.3 (1.9)	45.2 (3.7)	<0.001
Child’s weight, g	3,091 (359.1)	2,481 (281.6)	<0.001	3,065 (359.9)	2,329 (561.8)	<0.001
Gestational days at delivery, days	275.7 (10.1)	276.4 (11.3)	0.189	276.9 (7.9)	244.5 (18.3)	<0.001
Primiparous, %	1,465 (26.6)	107 (20.7)	0.003	1,522 (25.9)	57 (26.9)	0.753
Low birth weight (<2,500 g), % (%)	194 (3.5)	228 (44.0)	<0.001	294 (5.0)	130 (61.6)	<0.001
SGA, %				495 (8.5)	23 (10.9)	0.222
Stillbirth, %				—	—	
Preterm birth (<37 weeks), %	188 (3.4)	23 (4.4)	0.222	—	—	
Forced to change health check-up facility, %	1,927 (35.3)	174 (33.8)	0.496	2,059 (35.2)	71 (34.5)	0.822
Trimester of pregnancy at earthquake			0.360			0.179
First (2–14 weeks), %	1,765 (32.5)	166 (32.7)		1,888 (32.5)	69 (33.7)	
Second (14–28 weeks), %	2,181 (40.2)	190 (37.4)		2,313 (39.8)	91 (44.4)	
Third (≥28 weeks), %	1,483 (27.3)	152 (29.9)		1,605 (27.6)	45 (22.0)	
Infertility treatment, %	259 (4.7)	27 (5.2)	0.597	277 (4.7)	13 (6.1)	0.334
Placenta previa, %	85 (1.5)	4 (0.8)	0.165	76 (1.3)	13 (6.1)	<0.001^**^
Hypertensive disorders of pregnancy, %	156 (2.8)	40 (7.7)	<0.001	173 (2.9)	26 (12.3)	<0.001
Mental disorders before birth, %	344 (6.2)	24 (4.6)	0.145	360 (6.1)	12 (5.7)	0.791
Evacuation area, %	582 (10.6)	49 (9.5)	0.438	624 (10.6)	18 (8.5)	0.330

SGA, small for gestational age.

^*^t-test was used for continuous variables and chi-square tests was used for other categorical variables.

^**^Fisher’s exact test was used for other categorical variables.

Congenital anomalies were observed in 189 of 6,600 participants (2.9%). Infants with a congenital anomaly were smaller (mean 48.5; standard deviation [SD], 3.4 cm, vs mean 49.2; SD, 2.1 cm, *P* = 0.007), weighed less (mean 2,904; SD, 556.8 g vs mean 3,036; SD, 392.5 g, *P* = 0.002), had a shorter gestation period (mean 272.2; SD, 18.4 days vs mean 275.7; SD, 10.3 days, *P* = 0.013), and higher frequencies of preterm birth (12.6% vs 3.6%, *P* < 0.001), LBW (17.7% vs 7.2%, *P* < 0.001), and stillbirth (1.6% vs 0.1%, *P* = 0.001). These values increased among individuals “forced to change health check-up facility or intervals between visits due to disaster” (42.6% vs 35.2%, *P* = 0.038) and those with maternal mental disorders (9.5% vs 6.0%, *P* = 0.043).

LBW was observed in 445 of 6,561 participants (6.8%). LBW was less common in males (41.1% vs 51.9%, *P* < 0.001) and infants with LBW were shorter (mean 45.5; SD, 2.8 cm vs mean 49.5; SD, 1.8 cm, *P* < 0.001) and were born earlier (mean 260.6; SD, 18.9 days vs mean 277.0; SD, 8.3 days, *P* < 0.001) and was therefore more common in preterm births (30.7% vs 1.4%, *P* < 0.001). LBW also occurred more commonly in those treated for infertility (6.7% vs 4.5%, *P* = 0.030) and those with HDP (10.3% vs 2.7%, *P* < 0.001).

SGA was observed in 518 of 6,034 participants (8.6%). Infants with SGA were shorter (mean 47.1; SD, 2.3 cm vs mean 49.4; SD, 2.0 cm, *P* < 0.001), less commonly born to first-time mothers (20.7% vs 26.6%, *P* = 0.003), and more common among those with HDP (7.7% vs 2.8%, *P* < 0.001). Infant sex, gestational age at delivery, and preterm birth and infertility treatment rates did not differ significantly, whereas these values differed among those with LBW.

Preterm birth was observed in 212 of 6,111 participants (3.5%), and tended to occur in those with higher maternal ages compared to those of term births (mean 31.6; SD, 5.4 years vs mean 30.8; SD, 5.0 years, *P* = 0.040), and was associated with smaller infant height (mean 45.2, SD, 3.7 cm vs mean 49.3; SD, 1.9 cm, *P* < 0.001), lower birth weight (mean 2,329; SD, 561.8 g vs mean 3,065; SD, 359.9 g, *P* < 0.001), and increased LBW incidence (61.6% vs 5.0%, *P* < 0.001). Preterm birth was also more common in cases of placenta previa (6.1% vs 1.3%, *P* < 0.001).

Table 3 shows the results of binomial logistic regression analysis that determined the association between the occurrence of the primary outcomes of congenital anomalies, LBW, SGA, preterm birth, and maternal external radiation dose and factors associated with other outcomes.

**Table 3.** Associations between obstetric outcomes and external radiation dose (imputation of missing doses)

		Congenital anomaly						Low birth weight					
		Crude			Multivariate adjusted			Crude			Multivariate adjusted		
	Ref.	OR	(95% CI)	*P* ^*^	OR	(95% CI)	*P* ^**^	OR	(95% CI)	*P* ^*^	OR	(95% CI)	*P* ^**^
External radiation dose													
1 to <2 mSv	<1 mSv	0.81	(0.56–1.17)	0.253				0.91	(0.71–1.17)	0.448	0.91	(0.71–1.18)	0.472
≥2 mSv	<1 mSv	—	—					1.26	(0.56–2.83)	0.581	1.21	(0.53–2.79)	0.649
Maternal age	1SD	0.95	(0.82–1.10)	0.456				1.04	(0.94–1.14)	0.458			
Child’s sex	Female	1.20	(0.90–1.61)	0.221				0.65	(0.53–0.79)	<0.001	0.65	(0.53–0.79)	<0.001
Gestational days at delivery	1SD	0.79	(0.71–0.88)	<0.001				0.26	(0.23–0.29)	<0.001			
Primiparous	Multiparous	0.89	(0.63–1.26)	0.511				1.21	(0.98–1.50)	0.078			
LBW	≥2,500 g	2.78	(1.88–4.09)	<0.001				—	—				
SGA	≥−10%	1.67	(1.07–2.59)	0.023				21.6	(17.2–27.0)	<0.001			
Preterm birth	≥37 weeks	3.83	(2.40–6.11)	<0.001				30.5	(22.6–41.2)	<0.001			
Placenta previa	No	—	—					1.80	(0.95–3.40)	0.070			
Forced to change health check-up facility	No	1.36	(1.02–1.83)	0.038				1.02	(0.84–1.25)	0.830			
Trimester of pregnancy at earthquake													
Second (14–28 weeks)	First	0.78	(0.46–1.30)	0.335				1.12	(0.88–1.41)	0.363			
Third (≥28 weeks)	First	0.57	(0.31–1.05)	0.069				1.05	(0.81–1.37)	0.709			
Infertility treatment	No	1.31	(0.72–2.37)	0.376				1.54	(1.04–2.27)	0.031	1.49	(1.01–2.21)	0.046
Hypertensive disorders of pregnancy	No	0.84	(0.34–2.05)	0.695				4.18	(2.97–5.89)	<0.001	4.14	(2.93–5.84)	<0.001
Mental disorders before birth	No	1.66	(1.01–2.73)	0.045				0.89	(0.58–1.36)	0.592			
Evacuation area	No	0.99	(0.62–1.58)	0.959				0.91	(0.66–1.26)	0.573			

		SGA						Preterm birth					
		Crude			Multivariate adjusted			Crude			Multivariate adjusted		
	Ref.	OR	(95% CI)	*P* ^*^	OR	(95% CI)	*P* ^**^	OR	(95% CI)	*P* ^*^	OR	(95% CI)	*P* ^**^

External radiation dose													
1 to <2 mSv	<1 mSv	1.12	(0.91–1.40)	0.286	1.14	(0.92–1.42)	0.229	0.92	(0.65–1.30)	0.638	0.91	(0.65–1.29)	0.602
≥2 mSv	<1 mSv	0.84	(0.30–2.39)	0.744	0.84	(0.30–2.37)	0.735	1.08	(0.24–4.84)	0.919	1.05	(0.22–4.87)	0.955
Maternal age	1SD	0.93	(0.85–1.02)	0.105				1.17	(1.02–1.34)	0.025	1.13	(0.98–1.29)	0.084
Child’s sex	Female	0.87	(0.73–1.04)	0.134				1.28	(0.97–1.69)	0.081			
Gestational days at delivery	1SD	1.07	(0.98–1.18)	0.150				—	—				
Primiparous	Multiparous	0.72	(0.58–0.90)	0.004	0.69	(0.56–0.87)	0.001	1.05	(0.77–1.43)	0.753			
LBW	≥2,500 g	21.6	(17.2–27.0)	<0.001				30.5	(22.6–41.2)	<0.001			
SGA	≥−10%	—	—					1.32	(0.85–2.05)	0.222			
Preterm birth	≥37 weeks	1.32	(0.85–2.05)	0.222									
Placenta previa	No	0.50	(0.18–1.36)	0.174				5.01	(2.73–9.17)	<0.001	4.81	(2.60–8.89)	<0.001
Forced to change health check-up facility	No	0.94	(0.77–1.13)	0.496				0.97	(0.72–1.30)	0.823			
Trimester of pregnancy at earthquake													
Second (14–28 weeks)	First	0.93	(0.75–1.15)	0.490				1.08	(0.78–1.48)	0.650			
Third (≥28 weeks)	First	1.09	(0.87–1.37)	0.465				0.77	(0.52–1.12)	0.173			
Infertility treatment	No	1.12	(0.74–1.68)	0.597				1.33	(0.75–2.36)	0.334			
Hypertensive disorders of pregnancy	No	2.88	(2.01–4.12)	<0.001	3.01	(2.10–4.32)	<0.001	4.63	(2.99–7.17)	<0.001	4.50	(2.89–7.00)	<0.001
Mental disorders before birth	No	0.73	(0.48–1.12)	0.147				0.92	(0.51–1.67)	0.791			
Evacuation area	No	0.89	(0.65–1.20)	0.438				0.79	(0.48–1.28)	0.332			

CI, confidence interval; LBW, low birth weight; OR, odds ratio; SGA, small for gestational age.

^*^Univariate logistic regression analysis by forced entry method.

^**^Factors significant in univariate analysis were entered into multivariate analysis using binominal logistic regression.

Maternal external radiation dose (1 to <2 mSv) was not associated with congenital anomalies (OR, 0.81; 95% CI, 0.56–1.17, *P* = 0.253). Congenital anomalies, usually identified prenatally, may have necessitated a change of facilities after the disaster or caused mental disorders. Accordingly, post-disaster change of facilities and mental disorders were excluded from the binomial logistic regression analysis, despite being significant in univariate analysis.

Multivariate analysis of LBW was adjusted for dose (1 to <2 mSv, ≥2 mSv), sex (reference: female), infertility treatment, and HDP. The doses did not cause significant differences, with aORs of 0.91 (95% CI, 0.71–1.18, *P* = 0.472) for 1 to <2 mSv and 1.21 (95% CI, 0.53–2.79, *P* = 0.649) for ≥2 mSv. Male sex was independently associated with reduced LBW incidence (aOR 0.65; 95% CI, 0.53–0.79, *P* < 0.001), whereas infertility treatment (aOR 1.49; 95% CI, 1.01–2.21, *P* = 0.046) and HDP (aOR 4.14; 95% CI, 2.93–5.84, *P* < 0.001) were independently associated with increased LBW.

Multivariate analysis of SGA was adjusted for maternal radiation dose (1 to <2 mSv, ≥2 mSv), primiparity, and HDP. Different doses did not cause significant differences, and aORs of 1.14 (95% CI, 0.92–1.42, *P* = 0.229) and 0.84 (95% CI, 0.30–2.37, *P* = 0.735) for 1 to <2 mSv and ≥2 mSv, respectively, were observed. Primiparity was independently associated with reduced SGA (aOR 0.69; 95% CI, 0.56–0.87, *P* = 0.001), whereas HDP was independently associated with increased SGA (aOR 3.01; 95% CI, 2.10–4.32, *P* < 0.001).

Multivariate analysis for preterm birth was adjusted for dose (1 to <2 mSv, ≥2 mSv), maternal age, placenta previa, and HDP. The results did not differ significantly based on dose, and aORs of 0.91 (95% CI, 0.65–1.29, *P* = 0.602) and 1.05 (95% CI, 0.22–4.87, *P* = 0.955) for 1 to <2 mSv and ≥2 mSv, respectively, were observed. Placenta previa (aOR 4.81; 95% CI, 2.60–8.89, *P* < 0.001) and HDP (aOR 4.50; 95% CI, 2.89–7.00, *P* < 0.001) were independently associated with increased preterm birth.

Table 4 displays congenital anomaly types, of which the most common was cardiac malformation (0.86%). No congenital anomalies were found among the children born to mothers exposed to ≥2 mSv.

**Table 4.** Congenital anomalies (*n* = 6,600)

	Total	<1 mSv	1 to <2 mSv	≥2 mSv	(missing)
	*n* = 6,600	*n* = 2,188	*n* = 944	*n* = 54	*n* = 3,414
Total^*^	189 (2.86)	64	19	0	106
Cataract	1 (0.02)	0	1	0	0
Neural tube defects	3 (0.05)	1	2	0	0
Microcephaly	0 (0.00)	0	0	0	0
Cardiac malformation	57 (0.86)	20	4	0	33
Kidney/urinary tract malformation	19 (0.29)	5	3	0	11
Hydrocephaly	1 (0.02)	1	0	0	0
Cleft lip/palate	12 (0.18)	1	3	0	8
Digestive tract atresia	5 (0.08)	3	0	0	2
Imperforate anus	4 (0.06)	1	0	0	3
Poly/syndactyly	18 (0.27)	7	1	0	10
Others	83 (1.26)	28	6	0	49

^*^Multiple answers were allowed.

No outcomes were associated with external radiation exposure following analysis of missing external radiation dose information following multiple imputation. Similarly, we found no associated outcomes across all significant risk factors (Table 5).

**Table 5.** Association between obstetric outcomes and external radiation dose (no deficit in external radiation dose)

		Congenital anomaly						Low birth weight					
		Crude				Multivariate adjusted		Crude			Multivariate adjusted		
	Ref.	OR	(95% CI)	*P* ^*^	OR	(95% CI)	*P* ^**^	OR	(95% CI)	*P* ^*^	OR	(95% CI)	*P* ^**^
External radiation dose													
1 to <2 mSv	<1	0.64	(0.38–1.08)	0.096	0.64	(0.38–1.08)	0.096	1.04	(0.77–1.42)	0.791	1.06	(0.78–1.44)	0.720
≥2 mSv	<1	—	—					1.47	(0.58–3.75)	0.416	1.38	(0.53–3.55)	0.507
Maternal age	1SD	1.04	(0.84–1.29)	0.723				1.15	(1.00–1.32)	0.053			
Child’s sex	Female	1.14	(0.74–1.77)	0.558				0.67	(0.50–0.89)	0.005	0.67	(0.51–0.90)	0.007
Gestational days at delivery	1SD	0.73	(0.63–0.84)	<0.001				0.30	(0.25–0.35)	<0.001			
Primiparous, %	Multiparous	1.03	(0.63–1.69)	0.903				1.30	(0.96–1.76)	0.095			
LBW	≥2,500 g	3.22	(1.84–5.65)	<0.001				—	—				
SGA	≥−10%	1.30	(0.64–2.63)	0.469				26.3	(18.9–36.4)	<0.001			
Preterm birth	≥37 weeks	6.32	(3.36–11.88)	<0.001				25.1	(16.0–39.4)	<0.001			
Placenta previa	No	—	—					0.92	(0.28–2.98)	0.890		—	
Forced to change health check-up facility	No	1.74	(1.13–2.70)	0.013				1.08	(0.81–1.44)	0.620			
Trimester of pregnancy at earthquake													
Second (14–28 weeks)	First	0.78	(0.46–1.30)	0.335				1.01	(0.71–1.43)	0.974			
Third (≥28 weeks)	First	0.57	(0.31–1.05)	0.069				1.10	(0.76–1.58)	0.628			
Infertility treatment	No	1.28	(0.55–2.98)	0.566				1.59	(0.94–2.67)	0.083			
Hypertensive disorders of pregnancy	No	0.39	(0.05–2.80)	0.347				4.17	(2.54–6.83)	<0.001	4.11	(2.50–6.74)	<0.001
Mental disorders before birth	No	2.08	(1.06–4.09)	0.034				0.93	(0.51–1.69)	0.805			
Evacuation area	No	0.96	(0.49–1.87)	0.895				0.99	(0.64–1.51)	0.946			

		SGA						Preterm birth					
		Crude			Multivariate adjusted			Crude			Multivariate adjusted		
	Ref.	OR	(95% CI)	*P* ^*^	OR	(95% CI)	*P* ^**^	OR	(95% CI)	*P* ^*^	OR	(95% CI)	*P* ^**^

External radiation dose													
1 to <2 mSv	<1	1.26	(0.96–1.64)	0.092	1.27	(0.97–1.65)	0.083	1.05	(0.66–1.65)	0.844	1.04	(0.65–1.65)	0.875
≥2 mSv	<1	0.49	(0.12–2.02)	0.322	0.46	(0.11–1.92)	0.286	1.45	(0.34–6.11)	0.615	1.32	(0.30–5.77)	0.710
Maternal age	1SD	0.96	(0.85–1.09)	0.520				1.37	(1.11–1.70)	0.004	1.31	(1.06–1.62)	0.014
Child’s sex	Female	1.03	(0.80–1.32)	0.833				1.33	(0.87–2.04)	0.190			
Gestational days at delivery	1SD	0.99	(0.87–1.12)	0.827				—	—				
Primiparous, %	Multiparous	0.78	(0.58–1.05)	0.101				1.05	(0.66–1.68)	0.827			
LBW	≥2,500 g	26.26	(18.94–36.39)	<0.001				25.06	(15.95–39.39)	<0.001			
SGA	≥−10%	—	—					1.59	(0.85–2.95)	0.146			
Preterm birth	≥37 weeks	1.59	(0.85–2.95)	0.146				—	—				
Placenta previa	No	0.23	(0.03–1.64)	0.141				3.21	(1.13–9.16)	0.029	3.20	(1.10–9.33)	0.033
Forced to change health check-up facility	No	0.83	(0.64–1.09)	0.178				0.97	(0.62–1.51)	0.886			
Trimester of pregnancy at earthquake													
Second (14–28 weeks)	First	0.94	(0.69–1.28)	0.679				1.08	(0.66–1.75)	0.768			
Third (≥28 weeks)	First	1.05	(0.76–1.46)	0.750				0.61	(0.34–1.10)	0.101			
Infertility treatment	No	1.40	(0.85–2.30)	0.187				1.25	(0.54–2.91)	0.602			
Hypertensive disorders of pregnancy	No	2.92	(1.77–4.81)	<0.001	2.97	(1.80–4.91)	<0.001	6.11	(3.32–11.25)	<0.001	5.73	(3.09–10.63)	<0.001
Mental disorders before birth	No	0.57	(0.30–1.09)	0.089				0.70	(0.25–1.93)	0.489			
Evacuation area	No	0.90	(0.60–1.34)	0.597				0.68	(0.33–1.42)	0.304			

CI, confidence interval; LBW, low birth weight; OR, odds ratio; SGA, small for gestational age.

^*^Univariate logistic regression analysis by forced entry method.

^**^Factors significant in univariate analysis were entered into multivariate analysis using binominal logistic regression.

Although our analysis primarily included women who were pregnant at the time of the earthquake, Group B (13,020 individuals, excluding missing data on radiation dose [70.8%]; Figure 1) was subjected to the similar analysis. These results are presented in eTable 1.

## DISCUSSION

Our results revealed that 98.4% of participants were exposed to <2 mSv of radiation in the 4 months following the Great East Japan Earthquake, with a mean and maximum of 0.7 and 5.2 mSv, respectively. Major perinatal outcomes—LBW, SGA, preterm birth, and congenital anomalies—were not affected. LBW incidence decreased in males and increased in cases of infertility treatment and HDP. The frequency of preterm birth was elevated in cases of placenta previa and HDP.

Exposure was low, especially as evacuees of the Chernobyl accident exhibited an average internal and external exposure of 31 mSv.^28^ No effects on perinatal outcomes at low exposures were observed in surrounding countries following the incident.^29^ Associations with perinatal outcomes have been observed during ecological studies on FDND, but no direct association with actual exposure doses has been observed.^30^^–^^32^ These studies reported annual trends in the number of births, and possible worsening of perinatal outcomes, reduction in live birth rates, and a possible association between average prefecture-specific Cs-137 deposition and LBW were observed in radiation-contaminated prefectures. After the Chernobyl incident, residents experienced considerable internal exposure.^33^ However, food was strictly inspected for radioactive material following FDND, making significant internal exposure unlikely. A whole-body counter study found Cs-134 and Cs-137 internal exposure of residents of Fukushima Prefecture <1 mSv.^2^ The source of radiation exposure following FDND was γ-rays emitting from radioactive cesium in the environment. Current guideline recommends that fetuses not be exposed to >5 mSv.^34^ Most participants met this criterion. The incidence of perinatal outcomes in Fukushima Prefecture is comparable to that observed during the Japan Environment and Children’s Study (JECS), a nationwide birth cohort study encompassing the same period as this survey.^35^ The JECS lacks information on radiation exposure but surveyed 12,804 pregnant women during the same period in Fukushima Prefecture.

In our study, congenital anomalies were observed in 2.9% of cases, but only in 1.7% of participants in the JECS survey. The International Clearing House for Birth Defects Monitoring Systems Japan Center, which collects congenital anomaly statistics from all over Japan, reported an incidence rate of 2.4% nationwide in 2011.^36^ Our study was based on surveys completed by the mothers themselves, while the latter two studies were conducted using medical records. Fetal radiation exposure causes congenital anomalies at an estimated threshold of 100 mGy.^37^^–^^39^ The threshold for the development of microcephaly is 120 mGy or more.^40^ No participants reporting congenital anomalies were exposed to radiation doses >2 mSv. Radiation is evenly distributed over the body during maternal exposure, and the effective dose (mSv) can be directly replaced by the equivalent dose (mGy) for the fetus.^41^ Causes of congenital anomaly other than external exposure should be considered.

LBW was less common among males, while SGA was more common among first-time mothers, as previously reported.^42^^,^^43^ HDP was also an independent risk factor for SGA (<−1.5 SD) in the FHMS in FY2011.^8^ We adopted the standard international definition of weight <10th percentile for gestational age as SGA.^44^ Assisted reproductive technology increases the risk of LBW and SGA and is associated with preterm births.^45^^–^^47^ We defined fertility treatment as any mode of pregnancy besides spontaneous. We could not investigate the details of assisted reproductive technology in this study, which may have influenced the results.

HDP was observed in 3.3% of cases in this study but was observable in 2.5% of 12,804 cases evaluated during the JECS in Fukushima Prefecture (early onset, 0.3%; late onset, 2.1%),^35^ and is strongly associated to preterm birth.^48^

Placenta previa, which may require cesarean section (CS) due to sudden genital bleeding or causes massive bleeding^49^ during CS, is an acceptable cause of CS at <37 weeks gestation^50^ and was associated with preterm birth.

Indicators of physical and mental stress (“forced to change health check-up facility or intervals between visits due to disaster” and “mental disorders”) were not associated with any outcomes. Prenatal maternal stress in the fifth and sixth months of pregnancy increases the risk of LBW, SGA, and preterm birth.^51^ As we observed previously,^8^ no trimester was associated with perinatal outcomes for LBW or preterm birth, possibly due to the small sample size (versus 2.6 million in the literature).

This is one of the few epidemiological studies of pregnant women conducted over several years in a single region with support from a public institution. The strengths include the use of multiple imputation to clarify the results. Similar results were obtained through sensitivity analysis without multiple imputation conducted with information of those with maternal external radiation dose data. However, the results regarding the presence or absence of the main outcomes and survey variables may be inaccurate, as this survey was questionnaire-based. Another limitation is the lack of data on maternal body size or lifestyle (eg, smoking), which cannot be addressed in a survey of perinatal outcomes. The response rate of 60% may raise concerns about the representativeness of the target population; however, the factors associated with perinatal outcomes in this study were similar to those observed previously, so the results can be considered reliable. This survey confirms that maternal external radiation dose was low and not associated with the frequency of perinatal outcomes, which was a major concern of the people in Fukushima Prefecture. The results must be explained to pregnant and nursing mothers in Fukushima Prefecture. The impact of FDND on the perinatal outcomes remains a concern. The PBS aims to elucidate these outcomes and will continue to be conducted.

Maternal external radiation exposure following FDND was not associated with congenital anomalies, LBW, SGA, or preterm birth.

## SUPPLEMENTARY MATERIAL

eTable 1. Association between obstetric outcomes and external radiation dose in Group B
