# Supplementary material for: Effects of External Radiation Exposure on Perinatal Outcomes in Pregnant Women After the Fukushima Daiichi Nuclear Power Plant Accident: the Fukushima Health Management Survey
Source: J Epidemiol. 2022 Dec 5;32(Suppl 12):S104–14. doi: 10.2188/jea.JE20210252 (PMC9703922; doi:10.2188/jea.JE20210252)
Supplement: Supplementary file 1 [file je-32-S104-s001.pdf]

eTable 1. Association between obstetric outcomes and external radiation dose in Group B

		Congenital abnormality						Low birth weight					
		Crude			Multivariate adjusted			Crude			Multivariate adjusted		
	Ref.	OR	(95% CI)	*P**	OR	(95% CI)	*P***	OR	(95% CI)	*P**	OR	(95% CI)	*P***
External radiation dose													
1 to <2 mSv	<1	1.01	(0.80–1.28)	0.934	1.02	(0.80–1.29)	0.887	0.98	(0.86–1.12)	0.764	0.99	(0.86–1.13)	0.856
≥2 mSv	<1	1.00	(0.51–1.97)	0.998	1.02	(0.52–2.02)	0.945	0.82	(0.54–1.23)	0.332	0.82	(0.53–1.25)	0.347
Maternal age	1SD	1.11	(0.99–1.24)	0.082				1.04	(0.97–1.11)	0.259			
Child’s sex	Female	1.29	(1.03–1.62)	0.026	1.29	(1.03–1.62)	0.027	0.69	(0.61–0.79)	<0.001	0.68	(0.60–0.78)	<0.001
Days of from 3.11.2011	1SD	0.99	(0.89–1.11)	0.892				0.98	(0.92–1.05)	0.586			
Gestational days at delivery	1SD	0.79	(0.73–0.85)	<0.001				0.25	(0.23–0.27)	<0.001			
Primiparous, %	Multiparous	1.11	(0.88–1.39)	0.388				1.23	(1.08–1.40)	0.002	1.17	(1.03–1.34)	0.018
LBW	≥2,500 g	2.43	(1.82–3.24)	<0.001				–	–				
SGA	≥−10%	2.47	(1.74–3.50)	<0.001				23.22	(19.94–27.05)	<0.001			
Preterm birth	≥37 weeks	2.72	(1.92–3.84)	<0.001				25.39	(21.12–30.52)	<0.001			
Placenta previa	No	1.82	(0.85–3.91)	0.127				2.75	(1.85–4.10)	<0.001	2.46	(1.62–3.72)	<0.001
Infertility treatment	No	1.39	(0.96–2.01)	0.079				1.35	(1.08–1.67)	0.008	1.19	(0.94–1.49)	0.143

Hypertensive disorders of pregnancy	No	1.84	(1.14–2.95)	0.012	1.83	(1.14–2.93)	0.013	4.78	(3.82–5.98)	<0.001	4.63	(3.68–5.81)	<0.001
Mental disorders before birth	No	2.07	(1.35–3.17)	<0.001				1.26	(0.93–1.71)	0.141			
Evacuation area	No	0.71	(0.47–1.08)	0.107				0.89	(0.72–1.11)	0.300			
		SGA						Preterm birth					
		Crude			Multivariate adjusted			Crude			Multivariate adjusted		
	Ref.	OR	(95% CI)	*P**	OR	(95% CI)	*P***	OR	(95% CI)	*P**	OR	(95％ CI)	*P***
External radiation dose													
1 to <2 mSv	<1	1.07	(0.94–1.23)	0.292	1.08	(0.95–1.24)	0.250	0.94	(0.79–1.12)	0.480	0.95	(0.79–1.13)	0.557
≥2 mSv	<1	1.01	(0.68–1.49)	0.967	1.05	(0.71–1.55)	0.804	0.57	(0.30–1.07)	0.081	0.60	(0.32–1.15)	0.124
Maternal age	1SD	0.97	(0.91–1.04)	0.394				1.20	(1.10–1.31)	<0.001	1.17	(1.07–1.27)	<0.001
Child's sex	Female	0.92	(0.81–1.04)	0.187				1.15	(0.97–1.36)	0.099			
Days of from 3.11.2011	1SD	0.96	(0.90–1.03)	0.248				0.91	(0.84–0.99)	0.030	0.89	(0.82–0.97)	0.010
Gestational days at delivery	1SD	0.95	(0.89–1.01)	0.084				–	–				
Primiparous, %	Multiparous	0.85	(0.74–0.97)	0.014	0.82	(0.72–0.94)	0.004	0.96	(0.81–1.14)	0.659			
LBW	≥2,500 g	23.22	(19.94–27.04)	<0.001				25.39	(21.12–30.52)	<0.001			
SGA	≥−10%	-	–					1.85	(1.45–2.36)	<0.001			
Preterm birth	≥37 weeks	1.85	(1.45–2.36)	<0.001				–	–				

Placenta previa	No	0.75	(0.40–1.44)	0.389				6.38	(4.36–9.35)	<0.001	5.74	(3.87–8.53)	<0.001
Infertility treatment	No	1.14	(0.90–1.44)	0.281				1.37	(1.03–1.81)	0.030	1.09	(0.81–1.47)	0.563
Hypertensive disorders of pregnancy	No	3.06	(2.39–3.92)	<0.001	3.15	(2.46–4.04)	<0.001	5.78	(4.47–7.47)	<0.001	5.52	(4.25–7.18)	<0.001
Mental disorders before birth	No	1.22	(0.90–1.67)	0.207				1.52	(1.05–2.20)	0.026	1.45	(0.99–2.11)	0.057
Evacuation area	No	0.93	(0.75–1.14)	0.470				1.04	(0.80–1.36)	0.748			

CI, confidence interval; LBW, low birth weight; OR, odds ratio; SGA, small for gestational age.

* Univariate logistic regression analysis by forced entry method.

** Factors significant in univariate analysis were entered into multivariate analysis using binominal logistic regression.
